# Vericiguat: A New Horizon for Heart Failure Treatment

**DOI:** 10.31083/RCM48373

**Published:** 2026-04-20

**Authors:** Carla Carbonaro, Pier Paolo Bocchino, Eleonora Bertello, Carola Griffith Brookles, Filippo Angelini, Guglielmo Gallone, Stefano Pidello, Amedeo Feneziani, Claudia Raineri, Gaetano Maria De Ferrari

**Affiliations:** ^1^Department of Medical Sciences, University of Turin, 10124 Turin, Italy; ^2^Division of Cardiology, Cardiovascular and Thoracic Department, “Citta della Salute e della Scienza” Hospital, 10126 Turin, Italy

**Keywords:** vericiguat, heart failure, reduced ejection fraction

## Abstract

Despite advances in guideline-directed medical therapy (GDMT), heart failure with reduced ejection fraction (HFrEF) remains a progressive condition with high morbidity and mortality. Vericiguat, a soluble guanylate cyclase (sGC) stimulator, represents a novel therapeutic class that augments the nitric oxide–sGC–cyclic guanosine monophosphate (cGMP) pathway, which is impaired in HFrEF. The VICTORIA trial demonstrated that vericiguat significantly reduced the composite endpoint of cardiovascular death or heart failure hospitalization (HFH) in high-risk patients following a worsening event. Recent data from the VICTOR trial and subsequent pooled analyses suggest broader applicability, indicating that vericiguat may signal a potential mortality benefit in selected stable, ambulatory HFrEF patients with elevated natriuretic peptides but without recent hospitalization. The safety profile is favorable, with hypotension being the most common adverse event. Overall, vericiguat offers a valuable therapeutic option for a wide spectrum of HFrEF patients. Moreover, the ability of vericiguat to improve outcomes in both post-worsening and selected high-risk stable populations suggests this sGC stimulator may serve as a critical fifth component of GDMT, offering a new avenue for a personalized approach to HFrEF treatment. This review synthesizes key clinical evidence to elucidate the role of vericiguat in modern HFrEF management.

## 1. Introduction

Heart failure (HF) is a clinical syndrome defined by symptoms such as fatigue, dyspnea, and peripheral swelling, and by signs including elevated jugular venous pressure, pulmonary crackles, and peripheral oedema. It results from structural or functional cardiac abnormalities that lead to inadequate cardiac output and elevated intracardiac pressures [1].

The incidence of HF has declined in industrialized countries over recent decades, largely due to advances in cardiovascular prevention strategies. The estimated prevalence is between 1% and 2% in the general adult population, rising to more than 10% in individuals over 70 years of age [1, 2, 3].

The primary initiating factor in chronic HF is ventricular remodeling, which induces structural changes (dilatation, hypertrophy, fibrosis) and functional abnormalities (systolic and/or diastolic dysfunction) of the myocardium [4]. This results in an impaired ability of the myocardium to generate sufficient contractile force to maintain an adequate cardiac output, thereby failing to deliver the necessary blood and oxygen required to meet the metabolic demands of the body that result in hemodynamic instability, elevated filling pressures, renal dysfunction, and frequent decompensation. In advanced stages, HF is characterized by a progressive deterioration of myocardial structure and function, driven by maladaptive neurohormonal activation (renin–angiotensin–aldosterone system and sympathetic nervous system), endothelial dysfunction, oxidative stress, impaired nitric oxide (NO) signaling and reduced vasodilatory reserve which synergistically contribute to the worsening of the clinical syndrome and to adverse cardiovascular outcomes [5].

Over the last three decades, the management of heart failure with reduced ejection fraction (HFrEF) has undergone a paradigm shift. Several drug classes have proven capable of not only relieving symptoms but also reducing all-cause mortality and hospitalizations by modulating renin-angiotensin-aldosterone (RAAS) and sympathetic nervous systems. The current cornerstone therapy for HFrEF includes an angiotensin receptor–neprilysin inhibitor (ARNI) as first-line renin–angiotensin system blockade, or alternatively an angiotensin-converting enzyme inhibitor (ACEi) or angiotensin receptor blocker (ARB), in combination with a beta-blocker, a mineralocorticoid receptor antagonist (MRA), and a sodium–glucose cotransporter-2 (SGLT2) inhibitor [6, 7, 8, 9, 10, 11, 12, 13, 14, 15]. This “quadruple” therapy is recommended for patients with HFrEF featuring recommendation class I and evidence level A according to the current guidelines of the American College of Cardiology (ACC) and European Society of Cardiology (ESC).

Despite major therapeutic advances, HF remains a significant cause of morbidity, mortality, and healthcare utilization worldwide and quality of life is markedly reduced. Patients with HFrEF continue to experience worsening heart failure (WHF), leading to higher mortality risk and more frequent HFH [16]. This high-risk subgroup provided the rationale for exploring novel pharmacologic pathways beyond neurohormonal blockade and metabolic modulation.

These unmet needs underscore the necessity of novel therapeutic strategies targeting additional pathophysiological pathways beyond neurohormonal blockade. Among them, the nitric oxide (NO)–soluble guanylate cyclase (sGC)–cyclic guanosine monophosphate (cGMP) pathway has gained growing attention, providing the pharmacological rationale for vericiguat, a soluble guanylate cyclase stimulator. The NO–sGC–cGMP axis represents a fundamental regulatory pathway for cardiovascular and myocardial homeostasis and is profoundly disrupted in heart failure [17]. Decreased nitric oxide availability, together with oxidative impairment of sGC activity, results in reduced cGMP production, promoting vasoconstriction, pathological remodeling, and increased myocardial rigidity.

**Fig. 1.**
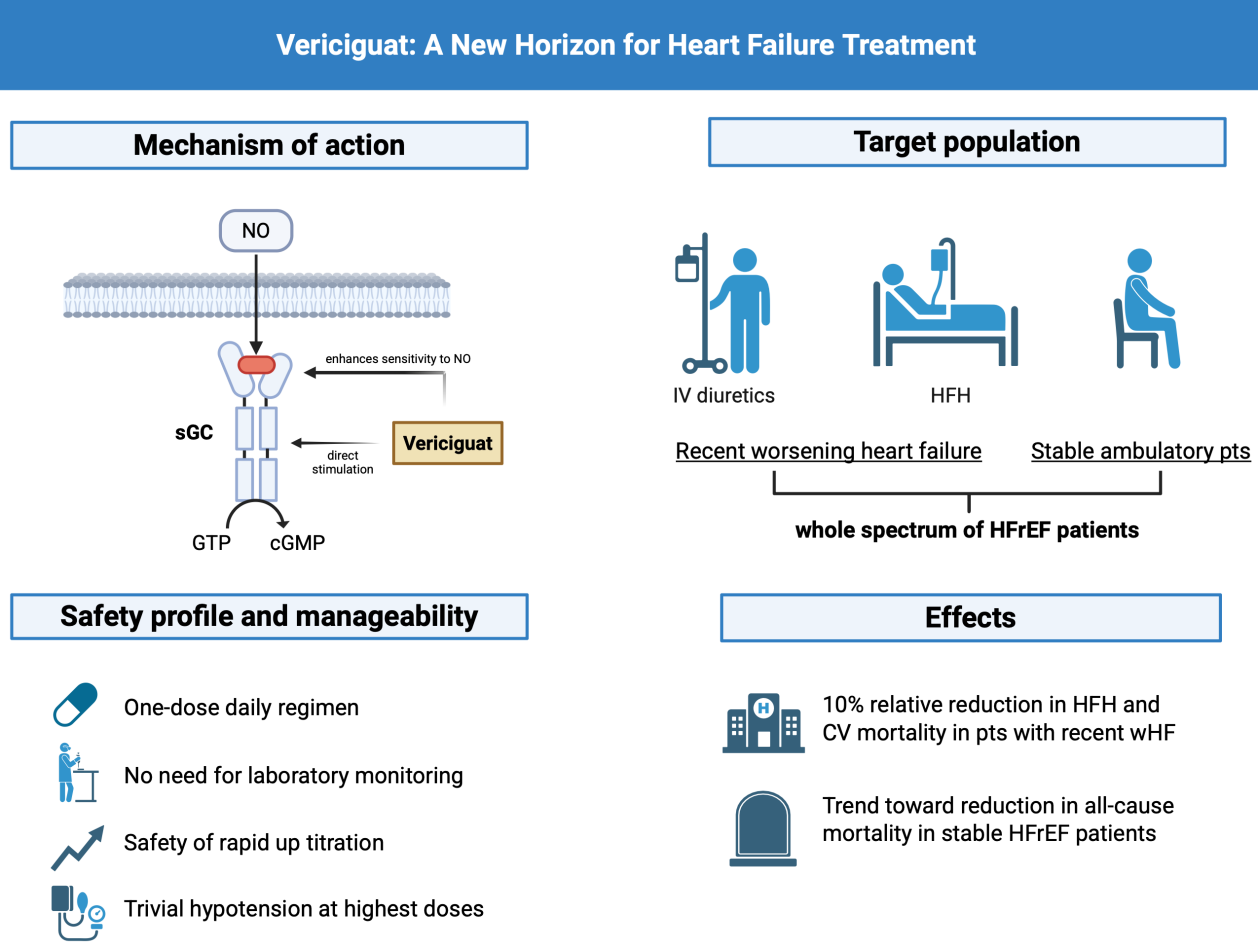
**Mechanism of action, target population, safety profile, and clinical effects of vericiguat in heart failure with reduced ejection fraction**. sGC, soluble guanylate cyclase; cGMP, cyclic guanosine monophosphate; NO, nitric oxide; HFH, heart failure hospitalization; GTP, Guanosine TriPhosphate; CV, cardiovascular. Figure created with BioRender.

Vericiguat is the first oral sGC stimulator to be approved to treat adults with symptomatic, chronic HFrEF and a recent history of worsening [1]. Vericiguat acts through a dual mechanism by directly activating native sGC in a NO-independent manner while simultaneously enhancing the enzyme’s sensitivity to residual endogenous nitric oxide [18]. This mechanism restores cGMP levels, activates protein kinase G, and promotes vasodilation, antifibrotic, and antihypertrophic effects—counteracting central pathophysiological processes of HF (Fig. 1). Recently, randomized trials have demonstrated that vericiguat reduces cardiovascular events and WHF in HFrEF [19].

The present narrative review aims to summarize current available evidence on vericiguat in patients with HFrEF, integrating mechanistic insights, clinical trial, and real-world data.

## 2. The Heart of the Matter: Physiopathology and Mechanism of Action

The normal vascular endothelium regulates vascular tone by balancing the production of vasodilators and vasoconstrictors, which regulate smooth muscle relaxation and contraction. Furthermore, the endothelium produces antiproliferative and anti-inflammatory cytokines that contribute to the maintenance of normal vascular function. NO is the predominant mediator of vasodilatation in the endothelium.

NO is synthesized through the conversion of L-arginine to L-citrulline catalyzed by endothelial nitric oxide synthase (eNOS). Owing to its gaseous nature, NO readily diffuses across cellular membranes, where it binds to the prosthetic heme group of sGC. This interaction induces the conversion of guanosine-5^′^-triphosphate to cGMP [20]. The latter represents a key second messenger that interacts with three types of intracellular proteins: cGMP-dependent protein kinases which mediate phosphorylation of downstream targets, cGMP-regulated ion channels which influence Ca^2+^ handling and contractility, and phosphodiesterases which regulate cGMP degradation and maintain signaling compartmentalization [21].

Through these effectors, the NO–sGC–cGMP pathway mediates multiple biological functions. In vascular smooth muscle, it promotes vasodilation by reducing cytosolic calcium concentrations and desensitizing myofilaments to calcium. In platelets, it inhibits aggregation, thereby exerting an antithrombotic effect. Within the myocardium, cGMP signaling contributes to both systolic and diastolic function by modulating calcium fluxes, improving relaxation (lusitropy), and enhancing compliance. Moreover, it regulates fibroblast activity, producing antifibrotic and antihypertrophic effects that limit pathological remodeling [22, 23, 24].

The release of NO is tightly regulated by shear stress, which arises from laminar blood flow [25].

Within the myocardium, NO regulates both contraction and relaxation by modulating calcium handling via S-nitrosylation of L-type calcium channels [26, 27]. The consequent decrease diminishes myofilament sensitivity to calcium, prolongs relaxation, enhances myocardial compliance, delays the onset of hypertrophy, induces vasodilation, and decreases vascular tension [28].

In the context of heart failure, this signaling cascade undergoes severe functional disruption. In patients with HFrEF, left ventricular pump dysfunction results in inadequate cardiac output and tissue hypoperfusion, which perpetuate systemic inflammation, oxidative stress, and neurohormonal activation. Collectively, these mechanisms act synergistically to markedly limit nitric oxide bioavailability [29, 30]. Reduced sGC activity is associated with coronary microvascular dysfunction, cardiomyocyte stiffness and interstitial fibrosis, elements that lead to the progression of myocardial dysfunction [22]. In advanced stages of HFrEF, sGC downregulation translates into diminished endothelium-dependent vasodilation, impaired coronary flow reserve, and progressive worsening of ventricular function [31].

Vericiguat belongs to a class of drugs known as sGC stimulators. It is approved for the treatment of patients with HFrEF and it acts via stimulation of sGC in the NO-sGC-cGMP pathway. Guided by extensive structure–activity relationship studies, vericiguat was developed as a next-generation derivative with enhanced pharmacokinetic properties, including greater metabolic stability and a prolonged half-life [29].

Vericiguat exerts a dual mechanism of action aimed at correcting the relative deficiency of cGMP observed in advanced stages of HF. First, it sensitizes sGC to endogenous NO by stabilizing the NO–sGC interaction. In addition, it directly stimulates sGC at a distinct binding site, independently of NO availability [32]. This selectivity in cyclic GMP generation does not occur with nitrates or phosphodiesterase inhibitors.

By combining these complementary actions, vericiguat increases intracellular cGMP levels within cardiomyocytes as well as vascular smooth muscle cells. By augmenting cGMP availability, vericiguat indirectly activates cGMP-dependent protein kinases that regulate pathways counteracting maladaptive processes such as cardiac hypertrophy and fibrosis [29]. Experimental models have shown that vericiguat attenuates oxidative stress triggered by angiotensin II stimulation, suppresses ERK signaling, downregulates fibrotic gene expression, and limits collagen deposition [33]. These effects translate into improved ventricular relaxation, increased myocardial compliance, and a slowing of diastolic dysfunction progression. In addition to its antifibrotic effects, sGC stimulators also attenuate excessive hypertrophy in response to pathologic stress. Through activation of protein kinase G (PKG), vericiguat interferes with upregulation of hypertrophic genes. PKG phosphorylates and inhibits L-type channels and TRPC-mediated entry, reducing cytosolic calcium and preventing calcineurin activation [34]. These effects are accompanied by improved systolic function, demonstrating that NO–sGC–cGMP signaling inhibits pathologic hypertrophy while maintaining compensatory adaptations. Furthermore, vericiguat exerts anti-inflammatory and immunomodulatory effects. Activation of the NO–sGC–cGMP cascade suppresses nuclear factor kappa-light-chain-enhancer of activated B cells (NF-κB) signaling by stabilizing Inhibitor of kappa B alpha (IκBα) and reduces Nucleotide-binding Oligomerization Domain (NOD), Leucine-Rich Repeats (LRR) and pyrin domain-containing protein 3 (NLRP3) inflammasome assembly, leading to a reduction in the release of pro-inflammatory cytokines (interleukin-1 beta (IL-1β), interleukin-18 (IL-18), tumor necrosis factor-alpha (TNF-α)) [35]. These effects contribute to limiting the inflammatory pathway that sustains adverse remodeling.

Beyond these molecular mechanisms, vericiguat exerts beneficial hemodynamic actions, most notably vasodilation and vascular smooth muscle relaxation, which together contribute to its therapeutic profile in HFrEF; in fact, agents with vasodilating properties are known to be beneficial in different settings of HFrEF [36, 37].

In addition to vasodilation and smooth muscle relaxation, vericiguat reduces systolic blood pressure (~1–2 mmHg) and HF biomarker *N*-terminal pro B-type natriuretic peptide (NT-proBNP) in a dose-dependent manner [38, 39].

## 3. A Winning Story: The Trials

First evidence regarding the use of vericiguat in patients with HF was provided by the SOCRATES (SOluble guanylate Cyclase stimulaToR in heArT failurE Study) program. This included two phase II, double-blind, dose-finding, placebo-controlled randomized clinical trials (RCTs), namely SOCRATES-REDUCED and SOCRATES-PRESERVED, which were completed in June and September 2015, respectively [38, 40]. Their primary objective was to evaluate the safety and tolerability of different daily dosages of vericiguat in patients with HF across the spectrum of left ventricular ejection fraction (LVEF). Both trials enrolled patients with chronic HF and recent clinical worsening, following stabilization of their cardiac condition.

The SOCRATES-REDUCED trial randomized 456 patients with LVEF <45% and New York Heart Association (NYHA) class II–IV who had received guideline-directed medical therapy (GDMT) for at least one month [38]. Eligible patients were those with recent WHF, defined by clinical signs or symptoms of congestion, elevated natriuretic peptides, or the need for hospitalization or intravenous diuretics, and subsequently achieved clinical stability with no requirement for intravenous diuretics within 12 hours or inotropes within 24 hours, and stable vital parameters. Randomization was performed either at hospital discharge or within four weeks after hospital discharge. Patients were assigned to receive placebo or one of four vericiguat dosing regimens: 1.25 mg or 2.5 mg (fixed dose), or 5 mg or 10 mg (titrated from a 2.5 mg starting dose). The primary endpoint was the absolute change in NT-proBNP levels at 12 weeks. Although the primary endpoint was not met, exploratory analyses suggested a dose–response relationship between vericiguat and NT-proBNP reduction, with the greatest benefit observed at the 10 mg dose. Improvements in LVEF were also noted at this dosage. No significant differences were observed between treatment groups in cardiovascular mortality or HFH. Importantly, vericiguat was generally safe and well tolerated. Episodes of hypotension were more common in patients receiving higher doses of vericiguat, but these events were largely confined to the first two weeks of therapy and rarely led to treatment discontinuation.

The SOCRATES-PRESERVED trial enrolled patients with HF and LVEF >45% who had experienced a recent worsening event (HFH or outpatient treatment with intravenous diuretics) [40]. The primary endpoints were changes in NT-proBNP levels and left atrial volume at 12 weeks. Although no significant differences were found between vericiguat and placebo for these endpoints, vericiguat demonstrated a favorable safety profile with low discontinuation rates and no significant effect on blood pressure, even at the highest dosages. Moreover, an exploratory analysis revealed that vericiguat at the dose of 10 mg daily was associated with improved quality of life, as assessed by the Kansas City Cardiomyopathy Questionnaire (KCCQ) clinical summary score. However, these results were not replicated in the following VITALITY-HFpEF randomized controlled trial [41]. This study enrolled 789 patients with similar features to those of the SOCRATES-PRESERVED trial: HF with LVEF >45%, elevated natriuretic peptides, NYHA class II-III and recent decompensation. Patients were randomized in a 1:1:1 ratio to receive either vericiguat (with a starting dose of 2.5 mg, later titrated up to 15 mg or 10 mg daily) or placebo, and were followed for 24 weeks. The study did not demonstrate any improvement in quality of life for the vericiguat group; this was assessed using the KCCQ physical limitation score and the six-minute walk test distance, which served as the primary and secondary endpoints, respectively.

Inspired by the promising results observed in the context of HFrEF, the phase III VICTORIA trial was conducted [42]. This double-blind, randomized trial enrolled 5050 patients with HFrEF (LVEF <45%), who were randomized to receive either vericiguat with an initial dose of 2.5 mg/day titrated up to 10 mg/day, or a placebo, both in addition to GDMT. Eligible patients had recently experienced clinical worsening, defined as HFH or treatment with intravenous diuretics, and were randomized after achieving clinical stability. Patients were stratified according to the timing of their worsening cardiac event: either HFH within the preceding 3 months, HFH between 3 and 6 months prior, or outpatient intravenous diuretic use within the preceding 3 months. Additional inclusion criteria included elevated natriuretic peptide levels, specifically BNP ≥300 pg/mL or NT-proBNP ≥1000 pg/mL in patients in sinus rhythm or BNP ≥500 pg/mL or NT-proBNP ≥1600 pg/mL in patients with atrial fibrillation. The primary endpoint was a composite of cardiovascular death or first HFH. The enrolled patients had a median age of 67 years, with 24% being female. Approximately 40% of the patients were in NYHA functional class III. The mean LVEF was 29%, and the median NT-proBNP level was 2816 pg/mL. Notably, two-thirds of patients were randomized within 3 months of hospitalization. Background therapy included triple GDMT in 60% of patients; notably, only 15% were receiving an ARNI and SGLT2 inhibitors were not yet part of standard care at the time of enrolment. After a median follow-up of 10.8 months, over 90% of patients in the vericiguat group were receiving the target dose of the medication. The incidence of the primary composite endpoint was significantly lower in the vericiguat group compared with placebo (hazard ratio [HR] 0.90; 95% confidence interval [CI] 0.82 to 0.98; *p*-value = 0.02), primarily driven by a reduction in HFH (HR 0.90; 95% CI 0.81 to 1.00). Subgroup analyses confirmed consistent efficacy across most patient categories, except for those over 75 years of age and those with NT-proBNP levels greater than 5314 pg/mL. Vericiguat was well tolerated, with no significant differences in the incidence of hypotension or syncope between the two groups.

Overall, the VICTORIA trial enrolled higher-risk patients than those enrolled in other clinical trials evaluating renin-angiotensin-aldosterone system inhibition or SGLT-2 inhibition in chronic HFrEF. This is demonstrated by higher baseline NT-proBNP values and a higher prevalence of NYHA class III-IV in the patients enrolled in the VICTORIA trial than those enrolled in the PARADIGM-HF, DAPA-HF and EMPEROR-reduced trials [9, 15, 43]. Vericiguat has thus been primarily tested and adopted as an add-on in post-worsening HFrEF, targeting patients with residual risk despite GDMT.

More recently, the efficacy of vericiguat was evaluated in ambulatory patients without recent worsening events in the Vericiguat Global Study in Participants with Chronic Heart Failure (VICTOR) trial [44]. This phase III, randomized, double-blind trial enrolled 6105 patients with HFrEF (LVEF <40%) who had not been hospitalized for HF in the preceding 6 months nor treated with intravenous diuretics in the 3 months prior to randomization. NT-proBNP levels at screening were required to be between 600 and 6000 pg/mL for patients in sinus rhythm and between 900 and 6000 pg/mL for those with atrial fibrillation. Patients with estimated glomerular filtration rate below 15 mL/min/1.73 m^2^ were excluded. Compared with those enrolled in the VICTORIA trial, patients included in the VICTOR trial were better treated: 56% were on ARNI, 58% on SGLT2-inhibitors, and 14.8% had cardiac resynchronization therapy. The median age of the study population was 68 years, the median LVEF was 30%, and the median basal NT-proBNP level was 1375 pg/mL; 79% of patients had NYHA II functional class. Nearly half of participants had no history of prior HFH. The primary endpoint was time to first occurrence of cardiovascular death or HFH, with cardiovascular death alone being a key secondary endpoint. After a median follow-up of 18.5 months, the target vericiguat dose was achieved in 85% of patients. No significant difference was observed in the primary endpoint between groups (HR 0.93; 95% CI 0.83 to 1.04; *p*-value = 0.22). The absence of a significant reduction in HF hospitalizations in VICTOR likely reflects the overall lower-risk profile of the enrolled population, characterized by the absence of recent decompensation events. Moreover, the broader uptake of SGLT2 inhibitors—agents that independently reduce HFH—may have further attenuated the relative treatment effect of vericiguat on this endpoint. Notably, in VICTOR the definition of worsening HF was broadened to include episodes managed with oral diuretic escalation, which did not confer a higher event risk and may have diluted treatment effect estimates.

Although fewer cardiovascular and all-cause deaths occurred in the vericiguat group (approximately 12% of patients receiving vericiguat compared with 14% receiving placebo), these results should be interpreted cautiously, since the primary study outcome was not met.

A pooled patient-level analysis of the VICTORIA and VICTOR trials evaluated the effect of vericiguat in patients with HFrEF across a broad spectrum of disease severity [45]. The VICTORIA trial enrolled high-risk patients with a recent worsening event, while the VICTOR trial included ambulatory, lower-risk patients without recent HFH. By combining these two largely non-overlapping populations, the analysis provided a comprehensive assessment of vericiguat’s efficacy. Treatment with vericiguat was associated with a significant reduction in the composite endpoint of cardiovascular death or HFH (HR 0.91, 95% CI 0.85 to 0.98; *p*-value = 0.0088). Significant effects were also observed on the individual components of the composite endpoint and on all-cause mortality, with no evidence of inter-trial heterogeneity. The therapeutic effect of vericiguat remained consistent across endpoints, independent of background treatment with ARNI or SGLT2 inhibitors. The therapeutic benefit appeared to be greater in patients with NT-proBNP levels below 6000 pg/mL. As previously reported, the VICTORIA trial enrolled a particularly high-risk population, in which HFH occurred three times more frequently than cardiovascular deaths, leading to an early accrual of events and trial completion. By contrast, the VICTOR trial enrolled ambulatory patients, many of whom were receiving quadruple guideline-directed therapy; in this lower-risk cohort, event rates were substantially lower, particularly among those without prior HFHs. Pooling the two trial populations therefore enables evaluation of vericiguat across the full clinical spectrum of HFrEF, suggesting a potential benefit when added to contemporary standard-of-care therapy.

To potentially simplify vericiguat titration and improve adherence compared to the multi-step approach used in prior studies, the Phase IIb, single-arm, open-label VELOCITY trial was designed to evaluate the safety and tolerability of a higher, single-step starting dose of vericiguat in patients with HFrEF [46]. The trial, which enrolled 106 patients, investigated whether initiating vericiguat at a 5 mg daily dose—bypassing the traditional 2.5 mg starting dose used in the VICTORIA trial—would be well-tolerated and safe. The primary endpoint, defined as completing a two-week period with no more than a one-day interruption and without moderate-to-severe symptomatic hypotension, was met by 93.4% of patients. This finding was consistent regardless of a recent history of WHF. By demonstrating the feasibility and tolerability of a 5 mg starting dose, the VELOCITY study supports an update in clinical practice to a simplified, one-step titration pathway for vericiguat, which could help overcome clinical inertia and facilitate more rapid achievement of the 10 mg target dose. In light of vericiguat’s favorable safety and tolerability profile, its once-daily dosing, and the lack of need for routine laboratory monitoring, the drug represents a feasible therapeutic option for patients with HFrEF in addition to current guideline-directed therapies. Nonetheless, these findings should warrant confirmation in future studies. The main characteristics of the trials on vericiguat are summarized in Table 1 (Ref. [38, 40, 41, 46, 47, 48]).

**Table 1.** **Main characteristics of the trials on vericiguat**.

Study	Sample size	Mean age	Female (%)	Mean LVEF	NT-proBNP thresholds	Median FU	Main findings
SOCRATES-REDUCED, 2015 [38]	456	68	19.7	29.6	≥1000 pg/mL	12 weeks	No significant change in NT-proBNP levels from basal to 12 weeks between vericiguat and placebo group.
							Exploratory analyses suggested a dose–response relationship between vericiguat and NT-proBNP reduction.
SOCRATES-PRESERVED, 2017 [40]	477	73	48	57	≥300 pg/mL (SR) ≥900 pg/mL (AF)	12 weeks	No significant change in NT-proBNP levels and LAV from basal to 12 weeks between vericiguat and placebo group.
							Pre-specified exploratory analysis showed a significant change in KCCQ-CCS from basal to 12 weeks in the vericiguat (10 mg) group compared to placebo.
VITALITY-HFpEF, 2020 [41]	789	73	48.7	56	≥300 pg/mL (SR) ≥900 pg/mL (AF)	24 weeks	No significant change in KCCQ-PLS from basal to 24 weeks between vericiguat and placebo group.
VELOCITY, 2025 [46]	106	67	28	/	No predefined cut-offs	2 weeks	Feasibility and good tolerability of vericiguat 5 mg starting dose, regardless of a recent history of WHF (93.4% of patients met the primary endpoint - completing a two-week period with no more than a one-day interruption and without moderate-to-severe symptomatic hypotension).
VICTORIA, 2021 [47]	5050	67	24	29	≥1000 pg/mL (SR) ≥1600 pg/mL (AF)	10.8 months	Significant reduction of the primary composite endpoint (CVd or first HFH) in the vericiguat group compared with placebo (HR 0.90; 95% CI 0.82–0.98; *p*-value = 0.02), primarily driven by a reduction in HFH. Subgroup analyses confirmed consistency across most categories, except for patients >75 years of age and those with NT-proBNP levels greater than 5314 pg/mL.
VICTOR, 2025 [48]	6105	68	23.6	30	≥600 pg/mL (SR) ≥900 pg/mL (AF)	18.5 months	No significant difference in the primary endpoint (time to first occurrence of CVd or HFH) between vericiguat and placebo group (HR 0.93; 95% CI 0.83–1.04; *p*-value = 0.22). Fewer CV and all-cause deaths occurred in the vericiguat group.

CI, confidence interval; CVd, cardiovascular death; FU, follow-up; HFH, hospitalization for heart failure; HR, hazard ratio; KCCQ-CSS, Kansas City Cardiomyopathy Questionnaire Clinical Summary Score; KCCQ-PLS, Kansas City Cardiomyopathy Questionnaire Physical Limitation Score; LAV, left atrial volume; LVEF, left ventricular ejection fraction; NT-proBNP, N-terminal pro B-type natriuretic peptide; WHF, worsening heart failure; HFpEF, Heart Failure with Preserved Ejection Fraction; SR, sinus rhythm; AF, atrial fibrillation.

## 4. The Real World Application: A Practitioner Textbook

Over the past decade quadruple therapy with ARNI, beta-blockers, MRA and SGLT2 inhibitors has become the standard of care in patients with HFrEF. In a network meta-analysis that compared different treatment strategies in the setting of HFrEF, the combination of these four drugs was found to be the most effective in reducing overall mortality [49]. Other treatment options involving the addition of agents such as vericiguat and ivabradine show benefit in specific patient populations, particularly in high-risk individuals who cannot tolerate quadruple therapy, most commonly due to worsening renal function and hypotension [50].

Currently, vericiguat is approved for the treatment of symptomatic HFrEF in several countries. The 2021 ESC HF guidelines recommend that vericiguat may be considered in symptomatic HFrEF patients with a recent worsening HF despite treatment with an ACEi (or ARNI), a beta-blocker and an MRA in order to reduce cardiovascular mortality and HFH (Class IIb, level of evidence B) [1]. Similarly, the 2022 AHA/ACC/HFSA guidelines for the management of HF suggest that vericiguat may be used in high-risk patients with HFrEF on top of optimal medical therapy after an episode of decompensated HF (Class IIb, level of evidence B) [51]. These recommendations are mainly based on the results of VICTORIA trial [42].

Secondary analyses of the VICTORIA trial suggest that the relative benefit of vericiguat is maintained regardless of the time elapsed since the WHF event [47]. However, the absolute benefit is greater when the index event is recent, thus supporting the early initiation of vericiguat in the post-discharge phase when patient vulnerability is at its peak. Nevertheless, prior to initiating vericiguat treatment, patients should first be stabilized following a WHF event by optimizing hemodynamics and volume status with appropriate diuretic therapy [52]. This approach minimizes risks of symptomatic hypotension and ensures tolerability of background therapy.

The evidence of the significant benefit of vericiguat from early trials has generated growing interest in the analysis of real-world data, which provides insights into patient characteristics, prescribing patterns, adherence, tolerability, and clinical impact outside the controlled environments of clinical trials.

An analysis of the PINNACLE Registry showed that approximately one-quarter of patients with HFrEF are eligible for vericiguat according to the VICTORIA trial criteria; also, the VICTORIA population was similar to PINNACLE patients with a recent WHF event regarding baseline characteristics and outcomes, thus suggesting that the patient population enrolled in the VICTORIA trial is likely generalizable to patients with WHF events encountered in clinical practice [53]. Similar data emerge from a Korean real-world study, where 58% of patients hospitalized for HF were found to be eligible for vericiguat therapy [54]. Data from the Swedish Heart Failure Registry show that one-fifth of patients are eligible for vericiguat according to the VICTORIA trial criteria, and approximately 47% based on guidelines and labelling. Thus, nearly half of the patients would be eligible if broader criteria were applied, suggesting that vericiguat could have a more extensive role in clinical practice [55].

Real-world studies indicate that patients treated with vericiguat are generally older, with more comorbidities, worse renal function, and more frequently on optimized quadruple therapy with ARNI, beta-blockers, MRA and SGLT2-inhibitors compared to the VICTORIA trial population [56, 57, 58]. In fact, the use of SGLT2 inhibitors was not recommended in the treatment of HF at the time of conduction of the VICTORIA trial, and only 15% of the enrolled patients were on ARNI therapy.

Based on the results of the VICTORIA trial, since 2021 the primary indication for vericiguat has been HFrEF with recent WHF event, in addition to GDMT. By 2025, the integration of vericiguat into clinical practice has matured, guided not only by the pivotal VICTORIA trial, but also by confirmatory data from the VICTOR trial, pooled analyses, and real-world experiences [45, 48]. These results represent emerging data that are not yet incorporated into current ESC 2021 or AHA/ACC/HFSA 2022 guideline recommendations and should therefore be interpreted as hypothesis-generating. A Japanese observational study has shown that in certain clinical contexts, vericiguat is used in high-risk patients even in the absence of a true worsening event [59]. The recently published VICTOR trial did not demonstrate a significant benefit in reducing the composite endpoint of cardiovascular mortality and HFHs in ambulatory patients with HFrEF who had not experienced a recent HFH, albeit showing a controversial benefit of vericiguat on cardiovascular mortality [48]. Likewise, the findings of pooled analyses of the VICTORIA and VICTOR trials, demonstrating the benefit of vericiguat across the broader HFrEF population regardless of recent HFH status, suggest that vericiguat may offer incremental benefits in reducing HFHs and cardiovascular mortality when added to standard GDMT in a broad HFrEF population [45].

A recent real-world analysis on a Japanese cohort of 46 patients suggests a potential role for vericiguat even in the absence of a recent decompensation episode, particularly in high-risk symptomatic patients who are not yet end-stage [60]. Patients with fewer than two prior HFHs showed greater improvements in biomarkers such as BNP and LVEF, along with a lower risk of adverse events. Conversely, patients with recurrent hospitalizations derived limited benefit, underscoring the importance of early intervention before irreversible disease progression occurs.

One of the most distinctive aspects of vericiguat’s clinical use is the emerging role of NT-proBNP as a stratification tool. In both VICTORIA and pooled analyses with VICTOR, treatment benefit appeared most pronounced in patients with NT-proBNP levels below 8000 pg/mL and even greater in patients with NT-proBNP levels below 4000 pg/mL. This finding suggests that physicians may consider prioritizing vericiguat for HF patients who, despite optimization of their medical therapy, have persistently elevated but not extremely high NT-proBNP levels, within a range where relative benefit is expected; this may represent a key patient subset in whom the drug’s efficacy is maximized. These observations suggest that vericiguat is a suitable treatment option for carefully selected high-risk patients who are not yet in the end-stage of heart failure [61].

The recommended starting dose of vericiguat is 5 mg orally once daily; if blood pressure is marginally low in the four weeks preceding treatment initiation, the starting dose should be 2.5 mg once daily. The dose should be doubled every two weeks to reach the target dose of 10 mg, if tolerated. Treatment should not be initiated in patients with systolic blood pressure below 100 mmHg. Discontinuation or dose reduction is advised in case of symptomatic hypotension or if systolic blood pressure falls below 90 mmHg. As food enhances the absorption of vericiguat, it is recommended that the drug is taken with meals; for patients who are unable to swallow, the tablet may be crushed and mixed with water immediately before administration [39, 52].

Since the VELOCITY study demonstrated the tolerability of an initial vericiguat dose of 5 mg/day, the standard starting dose of 2.5 mg may be bypassed in selected patients. Also, over 90% of patients safely tolerated vericiguat initiation at the dose of 5 mg/day, regardless of recent WHF events [46]. Accordingly, clinical guidelines now allow for a 5 mg starting dose in selected individuals, potentially simplifying the titration process, and facilitating the achievement of the target 10 mg dose in routine clinical practice.

Given its favorable safety and tolerability profile, vericiguat may be particularly useful in patients for whom titration of quadruple therapy is limited due to hypotension, bradycardia, renal impairment, or hyperkalemia. Its unique mechanism, acting via the NO–sGC-cGMP pathway, may aid in hemodynamic stabilization and facilitate the optimization of background therapy. Notably, a German observational study reported that the proportion of patients on full GDMT increased from 29% to 44% following vericiguat initiation [62]. Preliminary data also suggest a potential synergistic effect between ARNI and vericiguat, given their complementary actions on neurohormonal and vasodilatory pathways [63].

No dose adjustment is required in elderly patients or in those with mild to moderate hepatic or renal impairment. However, the effects of vericiguat have not been studied in patients with severe renal dysfunction (eGFR <15 mL/min/1.73 m^2^), severe hepatic impairment (e.g., Child-Pugh class C), or in those undergoing dialysis. Therefore, the initiation of vericiguat is not recommended in these populations [52].

Vericiguat has demonstrated a favorable safety profile in both randomized trials and observational studies. The most commonly reported adverse events were hypotension and anemia, which rarely required discontinuation of the drug [42, 48, 56, 62]. Co-administration with phosphodiesterase-5 inhibitors has not been studied in patients with HF, so it is not recommended due to the increased risk of symptomatic hypotension. The concomitant use of sGC stimulators such as riociguat is contraindicated [52]. There are no human studies on the use of vericiguat during pregnancy. Animal studies have shown potential fetal toxicity; therefore, vericiguat should not be used during pregnancy or in women of childbearing potential who are not using effective contraception.

Contemporary evidence, particularly from 2025 data, supports a paradigm shift in the use of vericiguat. This agent may be most effectively utilized earlier in the disease trajectory, including in carefully selected high-risk ambulatory patients who have not recently been hospitalized. This strategy aims to maximize therapeutic benefit before the patient progresses to an advanced or end-stage HF phenotype.

## 5. The Next Chapter: A Look Ahead

The landscape of HFrEF management continues to evolve toward a comprehensive, multimodal approach that targets not only neurohormonal activation but also endothelial dysfunction, oxidative stress, and myocardial remodeling. Within this paradigm, vericiguat has emerged as a promising therapeutic advance, expanding the armamentarium of disease-modifying drugs for HFrEF. The next decade will likely focus on refining its clinical positioning, identifying the optimal timing of initiation, and exploring new potential indications beyond current guideline recommendations.

From a clinical research perspective, several areas warrant further exploration. While the VICTORIA and VICTOR trials, together with their pooled analysis, established vericiguat as an effective and well-tolerated option across a broad spectrum of HFrEF severity [42, 44, 45], future studies should evaluate its role in patients with mildly reduced or preserved ejection fraction, where NO–sGC–cGMP signaling is also impaired [17]. Early-phase trials such as SOCRATES-PRESERVED [40] and VITALITY-HFpEF [41] failed to demonstrate significant improvements in clinical outcomes, yet preclinical data suggest that specific subgroups, particularly those with endothelial dysfunction or elevated natriuretic peptides, may still derive benefit. Precision phenotyping of these populations could redefine vericiguat’s therapeutic boundaries.

It should be noted that none of the available trials evaluated the potential synergistic effects of structured exercise training and a Mediterranean diet (MEDI) in combination with vericiguat therapy. Regular exercise training and adherence to a MEDI have been shown to improve endothelial function and NO bioavailability, thereby modulating the NO–sGC–cGMP signaling pathway and contributing to cardiovascular benefits [64, 65]; however, their potential interaction with pharmacological cGMP modulation has not been systematically evaluated in heart failure clinical trials.

Experimental studies have shown that vericiguat exerts antifibrotic, antihypertrophic, and anti-inflammatory effects by attenuating angiotensin II–induced oxidative stress [33], inhibiting the calcineurin–NFAT signaling pathway [34], reducing activation of NF-κB and NLRP3 inflammasome [35]. These findings support its potential as a disease-modifying agent capable of counteracting adverse myocardial remodeling.

In this context, additional data are needed to further investigate the hemodynamic and echodynamic impact of vericiguat, both in the short and long term. Prospective studies incorporating non-invasive hemodynamic monitoring, advanced echocardiographic indices (such as global longitudinal strain, E/e^′^ ratio, LVOT VTI), and right heart function assessment could shed light on the mechanisms underlying clinical benefits [66]. Such evidence would allow for a better understanding of vericiguat’s capacity to modulate ventricular-arterial coupling, reverse remodeling, and improve filling pressures—ultimately bridging the gap between symptom relief and structural recovery.

Clinically, vericiguat may be increasingly positioned for earlier initiation in high-risk ambulatory patients before the onset of advanced disease stages. Real-world data indicate that patients treated with vericiguat are often older, more comorbid, and already on full GDMT [56, 58]. However, the observed improvements in biomarkers, tolerability, and therapy optimization suggest that its early incorporation could yield incremental benefits. Owing to its favorable safety and tolerability characteristics, vericiguat emerges as an attractive therapeutic option. for patients in whom the optimization of quadruple GDMT is constrained by adverse hemodynamic or metabolic conditions—such as hypotension, bradycardia, renal dysfunction, or hyperkalemia. In such scenarios, vericiguat’s distinct mechanism of action through the NO–sGC–cGMP pathway may offer incremental benefits by stabilizing hemodynamics and facilitating the maintenance of background therapy. This approach aligns with a potential “penta-therapy” model—comprising ARNI, beta-blockers, MRA, SGLT2 inhibitors, and vericiguat—tailored to patient-specific clinical and hemodynamic profiles [50].

Despite the progress achieved, important questions remain. The long-term mortality benefit, cost-effectiveness in different healthcare systems, and safety in patients with severe renal impairment or advanced device therapy are still under investigation. The addition of another oral agent (vericiguat) to the treatment regimen may negatively impact patient adherence due to the already substantial pill burden common in the HFrEF population, who commonly require more than 5 daily medications; this increasing complexity may lead to treatment discontinuation. Moreover, future trials should clarify whether simultaneous versus sequential initiation with other GDMT components enhances outcomes and tolerability.

Ultimately, vericiguat represents more than a pharmacologic innovation—it symbolizes a conceptual shift toward restoring vascular homeostasis and myocardial resilience through the modulation of the NO–sGC–cGMP pathway [28]. As precision medicine advances, identifying the right patient at the right time for vericiguat initiation will be the key to unlocking its full therapeutic potential. By bridging the gap between hemodynamic stabilization and disease modification, vericiguat may help redefine the treatment paradigm of heart failure, guiding clinicians toward a more anticipatory, individualized, and pathophysiology-based management of this complex syndrome.

Within this evolving paradigm, attention has also been drawn to the potential role of circadian rhythms in cardiovascular regulation. Disruption of circadian rhythms has been associated with adverse cardiovascular outcomes, and experimental evidence suggests that restoration of circadian homeostasis may improve heart failure phenotypes. Although direct clinical data on chronotherapy targeting the NO–sGC–cGMP pathway are currently lacking, preclinical studies indicate that cGMP-dependent signaling is influenced by circadian mechanisms, providing a rationale for future investigations into time-dependent optimization of cGMP-modulating therapies [67].

## 6. Conclusions

Vericiguat represents a significant advancement in the contemporary treatment of HFrEF, addressing a residual pathophysiological domain not targeted by neurohormonal inhibition or metabolic modulation. By restoring cGMP bioavailability through direct and NO–dependent stimulation of sGC, vericiguat counteracts endothelial dysfunction, maladaptive remodeling, and progressive myocardial stiffening. Evidence from randomized controlled trials and real-world studies consistently supports the efficacy and safety of vericiguat, particularly in patients with recent worsening heart failure despite GDMT. The recent VICTOR trial and subsequent pooled analyses have expanded its potential role to stable ambulatory patients, suggesting a broader applicability across the HFrEF spectrum, while mortality benefits remain suggestive and primarily derived from pooled analyses.

Given its favorable safety profile, once-daily dosing, and mechanistic complementarity with existing pharmacologic agents, vericiguat may serve as a cornerstone of future HF management—an adjunctive “fifth pillar” that represents a therapeutic *continuum* from hemodynamic stabilization to disease modification. Its integration into clinical practice marks a transition toward a more individualized, pathophysiology-driven approach, reflecting the continuing evolution of HF therapy from symptom control to long-term preservation of myocardial integrity and patient survival.
